# Identification of Metabolic Phenotypes in Young Adults with Obesity by ^1^H NMR Metabolomics of Blood Serum

**DOI:** 10.3390/life11060574

**Published:** 2021-06-18

**Authors:** Khin Thandar Htun, Jie Pan, Duanghathai Pasanta, Montree Tungjai, Chatchanok Udomtanakunchai, Sirirat Chancharunee, Siriprapa Kaewjaeng, Hong Joo Kim, Jakrapong Kaewkhao, Suchart Kothan

**Affiliations:** 1 Center of Radiation Research and Medical Imaging, Department of Radiologic Technology, Faculty of Associated Medical Sciences, Chiang Mai University, Chiang Mai 50200, Thailand; ktdhtun28@gmail.com (K.T.H.); duanghathai.pa@gmail.com (D.P.); mtungjai@gmail.com (M.T.); chatchanok.u@cmu.ac.th (C.U.); siriprapa.k@cmu.ac.th (S.K.); 2 Shandong Provincial Key Laboratory of Animal Resistant Biology, College of Life Sciences, Shandong Normal University, Jinan 250014, China; 3 Department of Chemistry, Faculty of Science, Chiang Mai University, Chiang Mai 50200, Thailand; c.sirirat@gmail.com; 4 Department of Physics, Kyungpook National University, Daegu 41566, Korea; hongjoo@knu.ac.kr; 5 Center of Excellence in Glass Technology and Materials Science (CEGM), Faculty of Science and Technology, Nakhon Pathom Rajabhat University, Nakhon Pathom 73000, Thailand; jakrapong@webmail.npru.ac.th

**Keywords:** ^1^H NMR, young adults, obesity, hyperlipidemia, metabolic profile, metabolic syndrome

## Abstract

(1) Since the obesity prevalence rate has been consistently increasing, it is necessary to find an effective way to prevent and treat it. Although progress is being made to reduce obesity in the young adult population, a better understanding of obesity-related metabolomics and related biochemical mechanisms is urgently needed for developing appropriate screening strategies. Therefore, the aim of this study is to identify the serum metabolic profile associated with young adult obesity and its metabolic phenotypes. (2) Methods: The serum metabolic profile of 30 obese and 30 normal-weight young adults was obtained using proton nuclear magnetic resonance spectroscopy (^1^H NMR). ^1^H NMR spectra were integrated into 24 integration regions, which reflect relative metabolites, and were used as statistical variables. (3) Results: The obese group showed increased levels of lipids, glucose, glutamate, N-acetyl glycoprotein, alanine, lactate, 3 hydroxybutyrate and branch chain amino acid (BCAA), and decreased levels of choline as compared with the normal-weight group. Non-hyperlipidemia obese adults showed lower levels of lipids and lactate, glutamate, acetoacetate, N-acetyl glycoprotein, isoleucine, and higher levels of choline and glutamine, as compared with hyperlipidemic obese adults. (4) Conclusions: This study reveals valuable findings in the field of metabolomics and young adult obesity. We propose several serum biomarkers that distinguish between normal weight and obese adults, i.e., glutamine (higher in the normal group, *p* < 0.05), and lactate, BCAAs, acetoacetate and 3-hydroxybutyrate (higher in the obese group, *p* < 0.05). In addition, visceral fat and serum TG, glutamate, acetoacetate, N-acetyl glycoprotein, unsaturated lipid, isoleucine, and VLDL/LDL are higher (*p* < 0.05) in the obese with hyperlipidemia. Therefore, they can be used as biomarkers to identify these two types of obesity.

## 1. Introduction

Obesity is one of the greatest public health challenges of the 21st century. The rising global obesity rates have been substantial and have tripled since the 1980s in many countries based on the data from the World Health Organization (WHO) European Region [1,2]. Although Asian countries have some of the lowest prevalence of overweight and obesity worldwide, the alarming increase in overweight and obesity numbers in recent years has reached an epidemic level. Almost one billion people in Asia and the Pacific are overweight or obese. Central Asia has one of the highest rates of overweight and obese adults, but among those countries in the South East Asia region, Malaysia has the highest obesity prevalence at 14%, with Thailand next in line (8.8%) [3]. A majority of obese young adolescents will also have high potential to become obese as adults. As recent cross-sectional interventional data shows, it has been suggested that dietary fat composition and daily lifestyle are the main factors in excessive body fat accumulation, which could promote disease risk of metabolic syndromes such as insulin resistance and type 2 diabetes [4]. The scientific review of previous studies has reported strong associations between obesity and metabolic diseases [5,6]. 

Moreover, a comparison between overweight/obese (OW/OB) participants irrespective of their metabolic syndrome (MetS) status and normal-weight participants in childhood obesity has been revealed [7,8]. However, there has been no investigation of the relationship between serum metabolomic status, obesity, and lipidemic status in young adult subjects. Hyperlipidemia refers to high levels of low-density lipoproteins or triglycerides, which are the most commonly associated with obesity and diabetes. 

Non-hyperlipidemia obesity (NHLO) adolescents are obese, but they have no excessive lipid metabolic expression in the blood. The clinical correction for NHLO and hyperlipidemia obesity (HLO) may be quite different. Early screening for NHLO differentiation from HLO might help to refocus current prevention and treatment strategies and may even reduce healthcare costs. Although progress is being made to reduce young adult obesity, it is crucial to develop effective, targeted prevention and treatment strategies. Moreover, a detailed understanding of obesity-related biochemical alterations is urgently needed for the development of appropriate screening strategies. It is also important to trace the metabolic pathways involved in metabolic disorders and their association with metabolically active ectopic fat in the body.

In recent years, human-based metabolomics has emerged as an omics platform and is a powerful tool to study the underlying biochemical mechanisms and related metabolites disorders in obesity [9,10]. Pattern recognition methods integrate ^1^H NMR and mass spectroscopy (MS), together with multivariate statistical techniques. These are among the most widely applied analytical tools of metabolomics in the field of novel biomarkers. Metabolites have been discovered in a number of complex diseases, and thus can improve clinical diagnostics [11,12]. For example, Laura Brugnara et al. demonstrated how similar metabolic events attenuated the overall metabolic response in type 1 diabetes (T1D) patients after intense short-term exercise in both ^1^H NMR and gas chromatography mass spectroscopy (GC-MS) assayed studies done on the serum of the same subject samples [13]. The structural elucidation and functional characteristics/information of metabolites of interest are achieved by the interpretation of NMR spectral features, namely, chemical shifts and coupling constants once the sample is placed in a magnetic field [12]. Owing to the unique character of proton nuclear magnetic resonance (^1^H NMR) spectroscopy, its high reliability (coefficient of variation ~1–2%), excellent stability, superb integration accuracy, nondestructive, noninvasive analysis and the simultaneous detection of a broad range of metabolites from multicomponent mixtures makes it a feasible technique for metabolomic studies [14,15]. However, it has not yet been applied as a complementary metabolomic approach to investigate biochemical pathways associated with obesity in clinical practice. 

More recently, the combination of multivariate data analysis with high throughput ^1^H NMR spectroscopy tools are being used for identifying and quantifying small molecules in the biofluids such as serum and urine. This is being utilized for detection of biomarkers in biochemical and pathophysiological impairment in disease [7,16,17]. We aimed to evaluate the obesity-related metabolite alterations from normal weight subjects in order to identify the involved physiological pathways. Consequently, this was intended to develop the appropriate clinical biomarkers for exact determination of obesity related disease and assessment in the young population.

A non-targeted NMR-based metabolic profiling approach was applied to identify and quantify normal weight (NW) and OW/OB adolescent serum metabolites in this research. Consequently, this may help to trace the potential mechanisms by which body weight exerts pathological effects related to obesity and its associated hyperlipidemic complications. The main objective of this study was to investigate and compare serum metabolic profiles of obese and normal-weight young adults using an untargeted ^1^H NMR -based metabolomics approach. Moreover, the serum metabolic profiles of young NHLO and HLO adolescents were examined and compared. We aimed to determine possible metabolite biomarkers arising from obesity that have not been considered before.

## 2. Materials and Methods

### 2.1. Study Population

This prospective, cross-sectional study included 30 NW and 30 OW/OB volunteers aged 21.5 ± 0.95 years who were randomly chosen from a population residing in Chiang Mai, Thailand. Weight status was based on WHO guidelines [18]. NW has a BMI of 18.5–24.9 kg/m^2^ and OW/OB involves a BMI greater than or equal to 25 kg/m^2^. The subjects with a chronic disease, regular medication usage, and those that were athletes were excluded from the study using a questionnaire regarding health, lifestyle status and family medical history.

### 2.2. Ethical Considerations

The study was conducted following the Ethics Committee of the Faculty of Associated Medical Sciences, Chiang Mai University, Chiang Mai, Thailand (AMSEC-62EX-024). Informed consents were obtained from all participants. 

### 2.3. Biophysical Characteristics

The same observer measured every subject wearing examination cloth wear. Height (cm) and body weight (kg) were measured to the nearest 0.5 cm and 0.1 kg, respectively. Waist circumference (WC) (cm) and hip circumference (HC) (cm) were measured with a non-elastic tape while the subject softly exhaled. WC was measured at the midpoint of the distal border of the lower rib and the upper margin of the iliac crest (WHO guideline). HC was measured at the widest section of the buttocks. 

### 2.4. Conventional Biochemical Analyses

Blood collection was done by The Associated Medical Science Clinical Service Center, Chiang Mai University. Ten milliliters of intravenous blood were drawn from antecubital veins on the MR scanning day and was biochemically analyzed using a fully automated analyzer (Architect ci8200, Abbott Diagnostic). The test focused on total cholesterol (TC), low-density lipoprotein cholesterol (LDL-C), high-density lipoprotein cholesterol (HDL-C), triglycerides (TG), fasting blood sugar (FBS), glycated blood glucose (HbA1c), and alanine transaminase (ALT). Subjects were asked to fast for 10–12 h prior to blood examination. Later, LDL-C concentration was calculated from novel adjustable LDL estimation equations [19,20]. 

The National Cholesterol Education Project (NCEP) Adult Treatment Panel (ATP) III [21] has defined dyslipidemia as TC ≥ 200 mg/dL, TG ≥ 150 mg/dL, LDL-C ≥ 130 mg/dL, and HDL-C ≤ 40 mg/dL. Normal FBS ranges should be 70–100 mg/dL, and normal HbA1c levels should be less than 6% according to Diabetes care (2018). The current upper limit of serum ALT, though varied among laboratories, is generally around 40 IU/L [22].

### 2.5. Hyperlipidemia Obesity

Apart from the BMI, obesity-related hyperlipidemia determines the undesired amount of bad cholesterol in blood. Hyperlipidemia obesity is classified as being obese with TC ≥ 200 mg/dL, TG ≥ 150 mg/dL, and LDL-C ≥ 130 mg/dL. The group that fell in between the classification of non-hyperlipidemia obesity and hyperlipidemia obesity was termed metabolic dyslipidemia at-risk of obesity, but this group was not included in the statistical analysis. The obese with two or more of the above criteria were in the hyperlipidemia group. The obese whose lipid profiles did not relate to the above prescriptive hyperlipidemia values at all were denoted as non-hyperlipidemia obese [21,23].

### 2.6. Intraabdominal or Visceral Fat Composition Determined by MRI 

All volunteers underwent abdominal MRI by 1.5 Tesla, Ingenia, Philips MR machine (Philips Healthcare, Amsterdam, Netherlands) under comfortable conditions. The T1 weighted turbo spin-echo (TSE) (5 × 5 × 5 mm^3^) axial images of the abdomen with sense cardiac coil were collected. An axial slice at the L3-4 level was removed from the liver or buttock adipose tissue and saved using digital imaging and communications in medicine (DICOM) format for fat quantification. 

The image was analyzed using the Medical Image Processing, Analysis, and Visualization (MIPAV, National Institutes of Health, Bethesda, Maryland, MD 20814, USA) software package with a semiautomatic segmentation technique, which converts grayscale pixels into binary images of black and white, based on the signal intensity-based histogram-thresholding method [24]. The area with high signal intensity or that appears brighter represents adipose tissue and was set as the threshold to exclude the non-relevant organs and tissue in the image [25]. 

**Figure 1:**
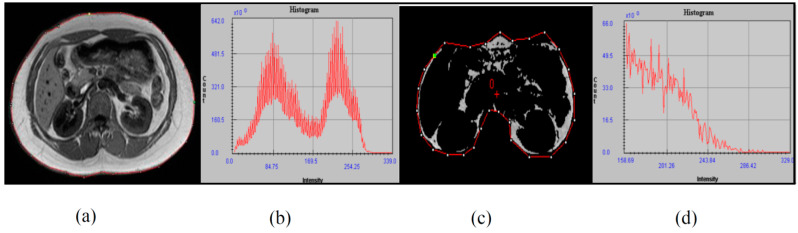
The image was analyzed using Medical Image Processing, Analysis, and Visualization-software package. (**a**) T1 weighted DICOM MRI image at the L3₋L4 level, (**b**) total abdominal tissue-related histogram, (**c**) segmented image for visceral adipose tissue, and (**d**) related histogram.

Pixels that appeared as white in the binary image represent adipose tissue content, while the black pixels represent soft tissue such as muscle, blood vessels, and bony structures, as shown in Figure 1. The visceral area was determined through manual drawing in the region of interest (ROI) and the abdominal wall separation between the intra-abdominal and extra-abdominal boundary [26]. After that, visceral fat percentage (Vis fat%) was calculated for total abdominal composition.

### 2.7. Sample Collection, Sample Preparation and ^1^H NMR Spectroscopy

Serum metabolites of NW and OW/OB groups were detected by ^1^H NMR method. Fresh serum samples were removed from −80 °C storage and thawed at room temperature. An optimized method was adopted for preparing the samples after centrifuging at 16,000× *g* for 10 min at 4 °C. Then, 100 μL of supernatant was dissolved by adding 500 μL of dimethyl sulfoxide-D6 with 99.8% deuteration mixed gently. The 550 μL of homogeneous solution was pipetted into 5 mm capillary tubes for NMR measurement. The proton spectrum was acquired at 27 °C on a Bruker AVANCE 500 MHz (Bruker, Bremen, Germany) with Carr-Purcell-Meiboom-Gill (CPMG, –RD–90°–(t–180°–t) n–acquire) using water-suppression pre-saturation pulse sequence. A 90° pulse with a 16 number of signal averaging (NSA) was applied. The baseline and phase correction were carefully adjusted by TopSpin 4.0.7 software. Spectra in the 0 to 8 ppm range were analyzed. The spectral data were normalized to the total integrated area prior to the data analysis. The metabolite resonances were assigned based on comparison with existing literature and human databases [27]. 

### 2.8. Statistical Analysis

After Kolmogorov–Smirnov test and Shapiro–Wilk test assessment, two-sample t statistics were performed as univariate analysis to determine the differences between NW and OW/OB and between NHLO and HLO groups according to biophysical profiles, biochemical results, and serum metabolites as detected by ^1^H NMR. The presented value was mean ± SD, and *p* < 0.05 denoted statistical significance. The relationship of fat composition, conventional biochemical, and ^1^H NMR metabolite profiles were verified by Pearson’s correlation analysis. Multivariate statistics were done using principal component analysis (PCA) to look for clustering and possible confounders in the data set. Next, partial least squares discriminant analysis (PLS-DA) was used in order to train a model (statistical classifier) to discriminate between NW and OW/OB groups, and between NHLO and HLO. The validity of the established models was shown by the total amount of variation between the groups explained as R2Y (cum %) and within the group denoted as R2X (cum %), respectively. The predictive ability of the model was determined by cross validation and denoted as Q2 (cum %). The variable with significant difference in univariate analysis (Two sample *t* test) and PLS-DA variable importance for the projection (VIP) value exceeding 1 was defined as the strongest variable contributing to group discrimination. Statistical analysis was performed using IBM SPSS version 17 and OriginPro 2015.

## 3. Results

### 3.1. Anthropometric and Clinical Characteristics

The descriptive biophysical, biochemical characteristics of the study population are presented in Table 1. The body weight, BMI, HC, and WC increased significantly in the OW/OB group with *p* < 0.001, compared with NW adolescents. 

According to biochemical lipid profiles results, TC, LDL-C, and TG level elevations in OW/OB were significantly different from NW with *p* < 0.05 and *p* < 0.001, respectively. Blood TG had the highest degree of significant changes between OW/OB (115.23 ± 43.89 mg/dL) and NW (72.5 ± 13.25 mg/dL). There was an obvious downregulation of HDL-C lipid seen in the OW/OB group compared to NW. The glycemic determinants FBS and HbA1c levels were in the normal range, but they were distinctly higher in OW/OB than the NW group (89.45 ± 5.21 mg/dL vs. 82.97 ± 2.71 mg/dL) and (5.19 ± 0.17% vs. 5.01 ± 0.195%). The ALT increase was accompanied with a TC increment in OW/OB, seen in Table 1. 

OW/OB occupied about 11.30 ± 4.31% of the visceral fat compartment, which was about 3.5 times higher than that in NW. 

### 3.2. ^1^H NMR Metabolomic Characteristics of NW Versus OW/OB Adolescents

**Figure 2:**
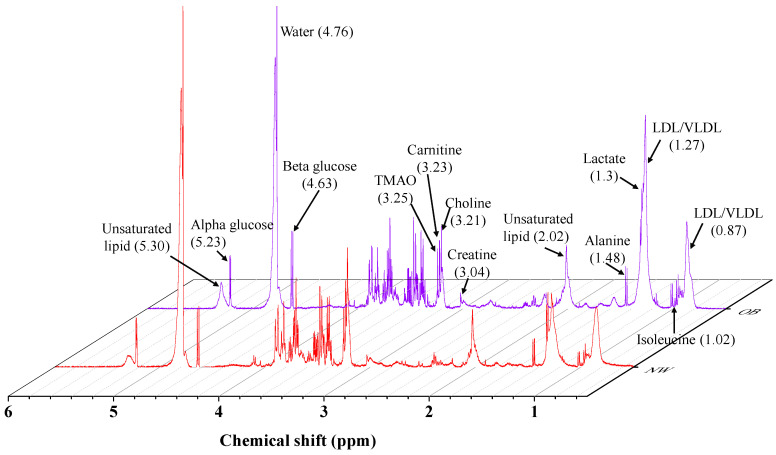
Overlapping of ^1^H NMR serum spectra of NW in red and OW/OB in blue color.

A zoom-in of a typical ^1^H NMR spectra overlaying of NW and OW/OB subjects is illustrated in Figure 2. The metabolite differences and the changing trend between NW and OW/OB subjects are presented in Table 2. For the metabolic profiling, multivariate PLS-DA statistics were used to train a classification model (classifier) to discriminate between NW and OW/OB controls based on data input from their metabolic profile. In other words, the integration values of the 24 regions (metabolic profiles) were recognized from blood serum ^1^H NMR spectra related to differential BMI intervention. 

**Figure 3:**
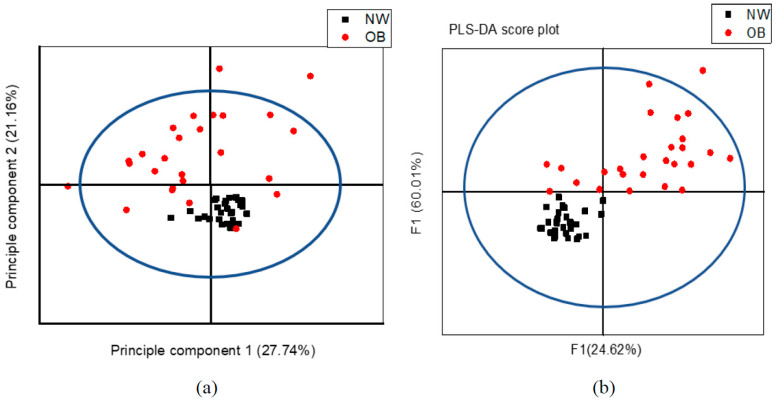
^1^H NMR variables of 30 NW in black and 26 OW/OB in red, are plotted according to different BMIs based on the 24 sera, (**a**) PCA score plot and, (**b**) PLS-DA score plot.

A PCA analysis was done first to look for clustering, possible confounders, and outliers. The individuals illustrated on the PCA score plots each represented the 24 variables obtained from its ^1^H NMR spectrum, which are clustered in a way that allows OW/OB individuals to be clearly differentiated from their NW counterparts. This model explained 27.74% of variation according to principal component 1 (PC1) as the largest variance within the data set and 21.16% of variation according to principal component 2 (PC2) as the second-largest variance within the data. The PCA score plot shows clustering of observations when colored according to BMI status as shown in Figure 3a. 

Multivariate PLS-DA was used to train a classification model (classifier) in discriminating between the 26 OW/OB and 30 NW individuals based on data input from their metabolic profile. The model explained that the total amount of variation within the groups was 54.20% [R2X (cum)] and 77.63% [R2Y (cum)] between the groups. The model’s predictive ability was good enough to discriminate between the groups based on the metabolic profile with a Q2 (cum) of 60.37%. Figure 3b, a PLS-DA score plot, shows the first predictive component (F[1]P: 24.62%), explaining the variation between the groups, versus the first orthogonal component (F[1]O: 60.01%) that explains the variation within the groups. Between NW and OW/OB individuals, according to univariate significance (*p* < 0.05) and PLS-DA VIP value >1, unsaturated lipid CH=CH (5.3 ppm), glutamate (2.34 ppm), unsaturated lipid =CH_2_ (2.02 ppm), lactate (1.3 ppm), (-CH_2_)_n_ VLDL/LDL (1.27 ppm), and isoleucine (1.02 ppm) were significantly higher, whereas choline (3.21 ppm) was lower in OW/OB with respect to NW. The most significant change in serum concentration between OW/OB and NW adolescents is observed at variable 11, representing glutamate and variable 18 representing saturated lipid (-CH_2_)_n_ VLDL/LDL, shown in Table 2.

Pearson’s correlation of biochemical lipid profiles with ^1^H NMR lipid biomarkers was significantly associated with different associations. HbA1c% and FBS were obviously related to glycolysis metabolites, beta-glucose, total glucose, and lactate levels in ^1^H NMR results with the moderate correlation coefficient values shown in Table 3 and Table 4.

There was a significant and moderate correlation of those seven dominant metabolites to visceral fat composition, as shown in Table 5. Except for choline, the other six metabolites were positively related to visceral fat. Unsaturated lipid =CH_2_ was the strongest associated triglyceride with visceral fat (r = 0.656, *p* < 0.001) among them.

### 3.3. ^1^H NMR Metabolomic Characteristics of NHLO Versus HLO Adolescents

The OW/OB group was further subdivided into 13 NHLO and 13 HLO adolescents. Overweight/obese participants who could not be defined as NHLO or HLO were classified as metabolically at-risk obese (*n* = 4) and were not included in the statistical analysis. No statistical differences were found for age, weight, BMI, and WC between NHLO and HLO subjects. Only TC, LDL-C, and TG differences were found in the HLO phenotype as shown in Table 6. The obese hyperlipidemia group had TC = 238.77 ± 33.67, LDL-C = 160.18 ± 32.12, and TG = 138.69 ± 42.17, respectively. These values were the only significant increments in the HLO group compared to NHLO group.

**Figure 4:**
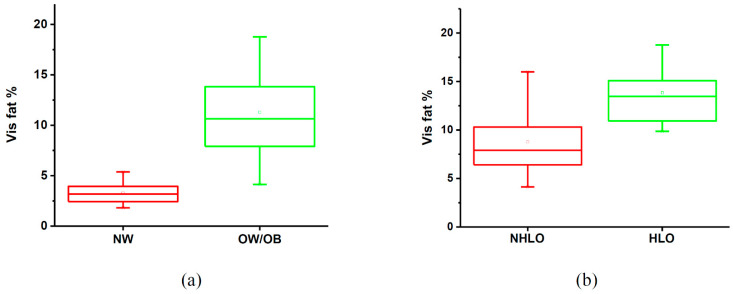
Box plot of mean visceral fat percentage, (**a**) normal-weight (NW) and overweight/obese (OW/OB), (**b**) non-hyperlipidemia obesity (NHLO) and hyperlipidemia obesity (HLO).

The remaining biophysical and biochemical characteristics were not significantly different at all as shown in Table 6. Furthermore, NHLO had less visceral lipid than the HLO group, at 8.77 ± 3.29%. A comparison box chart is shown in Figure 4. The metabolite differences and the changing trends between NHLO and HLO subjects are presented in Table 7.

**Figure 5:**
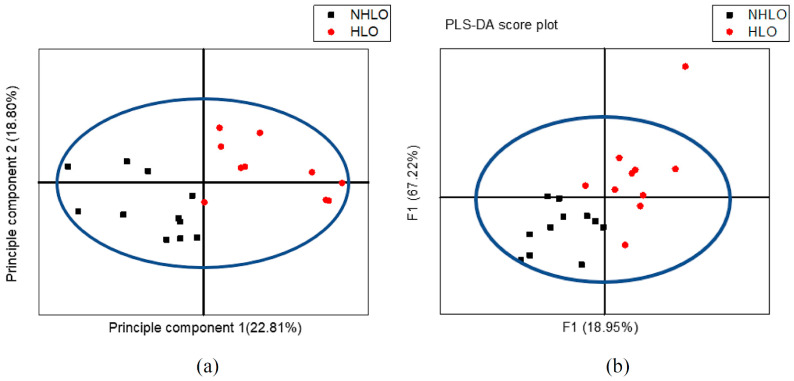
^1^H NMR variables of 13 non-hyperlipidemia (NHLO) obese in black color and 13 hyperlipidemia obese (HLO) in red color are plotted according to lipid profile parameters based on the 24 sera, (**a**) PCA score plot, and (**b**) PLS-DA score plot.

Regarding metabolic profiling, the PCA score plot shows a clear differentiation between NHLO and HLO individual clusters in Figure 5a. Moreover, the PCA model is confounded by lipid. This model explained 22.81% of variation according to principal component 1 (PC1) as the largest variance within the data set and 18.80% of variation according to the principal component 2 (PC2) as the second-largest variance within this data. An OPLS-DA model (statistical classifier) was trained to differentiate between the 13 NHLO and 13 HLO individuals. Figure 5b explains the total amount of variation within the groups of 86.92% [R2X (cum)] and between the groups of 99.8% [R2Y (cum)]. The model still has an average predictive ability of 74.33% [Q2 (cum)]. The PLS-DA score plot shows the first predictive component (F[1]P: 18.95%), explaining the variation between the groups, versus the first orthogonal component (F[1]O: 67.22%) that also explains the variation within the groups as seen in Figure 5b. Between NHLO and HLO individuals, according to univariate changes (*p* < 0.05) and PLS-DA VIP value >1, glutamate (2.34 ppm), acetoacetate (2.22 ppm), N-acetyl glycoprotein (2.14 ppm), unsaturated lipid =CH_2_ (2.02 ppm), isoleucine (1.02 ppm) and (-CH_3_) VLDL/LDL (0.87 ppm) were significantly higher in HLO compared to NHLO. The most significant change in serum concentration between HLO and NHLO adolescents is observed at the variable 12, representing acetoacetate at 45.45%, as shown in Table 7.

## 4. Discussion

Recently, there has been increases in the prevalence of obesity among children and adolescents, and this has become a major public health problem worldwide [3]. There needs to be an in-depth understanding of the association between obese mechanisms and comorbidities, yet there are still no reliable biomarkers to indicate metabolic alterations associated with obesity and other complications. Integrated platforms have been frequently employed to provide sensitive and reliable detection of thousands of metabolites in samples of bio-fluids instead of applying a single analytical platform to identify the biological relevant pathways and pathophysiologic factors [28].

Metabolite studies on both animals and humans have explored potential biomarkers and underlying biological mechanisms of obesity and correlations with metabolic activity [29,30]. Yanpeng An el al.investigated the data of NMR analysis using observations from GC FID-MS techniques for fatty acid compositions in cases of HFD-caused metabonomic changes in two biological matrices of rat liver and blood plasma [31]. Cases regarding the application of NMR-based metabolomics on the obese adolescent population are almost entirely unexplored. The present study demonstrates that obese and normal-weight adolescents can be clearly distinguished by using means of untargeted NMR-based serum metabolomics. The obese serum metabolome is characterized by higher concentrations of lipids triglycerides, glutamate, lactate, and isoleucine, and lower concentrations of chlorinated phospholipids as compared with the normal-weight serum metabolome.

Obese children and adolescents are often distinguished with dyslipidemia characterized by higher TC, the greater presence of low-density LDL particles, and low HDL-C concentrations from normal-weight subjects [32]. Our study shows that serum lipid levels (saturated and unsaturated fatty acid chains of triglycerides) are increased in obese compared with normal-weight adolescents. Among them, variable number 14 mainly represents the CH_2_ protons of unsaturated fatty acid chains, and 18 represents CH_2_ protons of saturated fatty acid chains that are found to be a tendency in lipid metabolites of obese groups. Those fatty acid chains of triglycerides in the serum NMR study revealed a significant linear correlation of TC, TG, and LDL laboratory results. Cali et al. concluded that the normal-glucose obese adolescents with hepatic steatosis have higher hepatic production and secretion of triglycerides containing VLDL particles in the blood, which are subsequently converted to LDL [33]. In fact, a more pronounced visceral fat depot was found in obese adolescents with fatty livers than their matched controls. In addition, a strong association between fatty liver and visceral adiposity in adults has been reported [34]. Although our data does not include hepatic fat information, which refers to a portal hypothesis, free fatty acid fuel for the hepatic lipogenesis process may be obtained from high metabolic visceral fat lipolysis pathways. Our results indirectly highlighted that hepatic overproduction of VLDL is related to greater visceral fat deposition rate that constitutes the metabolic basis of various hyperlipidemic states in humans, such as obesity and is also associated with hyperlipidemia.

Furthermore, our study shows some noticeable metabolite changes taking place in OW/OB compared to the NW group. Although the NMR glucose tributes changes were not as high as the biochemical results (FBS and HbA1c) in this study, a substantial upward trend of lactate with VIP >1 was seen. Lactate is one of the substances produced by cellular metabolism when the body breaks down carbohydrates for energy synthesis when the oxygen level is low. Our study likely points to a more significant fat mass induced lipid accumulation in the adipocyte, and those low-mitochondria hypertrophic adipocytes activate the anaerobic glycolysis pathway instead of acetyl CoA conversion through aerobic metabolism [35]. In obese humans, 50–70% of all glucose is metabolized by the large adipocytes in the conversion of glucose to lactate [35,36]. Furthermore, the lower vasculature of enlarged adipocytes also induces local adipose tissue hypoxia. The release of higher lactate represents the cells being deprived of adequate oxygen supply [37]. In the present study, greater amounts of lower vasculature and enlarged adipocytes characteristic of visceral white adipose tissue in OW/OB may cause a more pronounced release of lactate in the circulation compared to NW with considerable increases occurring by an estimated 26%. This demonstrated that lactate seems to be a possible discriminate biomarker in obesity determination.

The most abundant phospholipid class contained in lipoproteins is phosphocholines. It is the primary source of choline in the body. Phosphatidylcholine or phosphocholine synthesis by the phosphatidyl-ethanolamine N-methyltransferase (PEMT) pathways is required for VLDL assembly in the liver and then transports it into the bloodstream to extrahepatic tissues. Approximately 70–95% of VLDL phospholipids are phosphatidylcholines in humans, and inadequate phosphatidylcholine synthesis induces more fat and cholesterol accumulation in the liver [38]. We found that the choline metabolite was significantly decreased in OW/OB compared to NW and inversely proportional for subjects having more significant visceral fat. Obesity caused disorders in choline metabolism and there was also decelerated choline activity in the obese. It is the consensus that choline can promote the adipose tissue hydrolysis rate, and the decreased choline content may eventually induce nonalcoholic fatty liver, which is a vital determinator of obesity [39].

Choline is a compound in liver tissue, which has an important effect on the synthesis and transport of lipids. It was identified as a basic nutrient in 1998. Choline protects the liver by promoting the secretion of ultra-VLDL (an essential component for exporting fat to the liver) in the liver. It is known that choline and methionine deficiency causes decreased synthesis of phosphatidylcholine, and consequently the secretion of VLDL is blocked, triglycerides out of the liver are blocked, and finally hepatic steatosis is caused. In the present study, we found that the choline metabolite was significantly decreased in OW/OB compared to NW and was inversely proportional for subjects having more significant visceral fat. Obesity caused disorders in choline metabolism and there was also decelerated choline activity in the obese. Choline was lower in OW/OB when compared to NW (0.049 ± 0.011 vs. 0.068 ± 0.0090, at 3.21 ppm, *p* < 0.001), and it is significantly decreased in HLO vs. NHLO (0.043 ± 0.005 vs. 0.054 ± 0.012, *p* < 0.05). It is the consensus that choline can promote the adipose tissue hydrolysis rate, and the decreased choline content may eventually induce nonalcoholic fatty liver, which is a vital determinant of obesity [39].

Choline is the precursor of acetylcholine (Ach); Ach is synthesized by choline and acetyl-CoA under the catalysis of choline acetyltransferase (choline acetylase) and can be metabolized by cholinesterase into choline and acetic acid. Choline in food is taken into cells through the choline transporter CTL1 and is metabolized by choline kinase, which converts it into phosphorylcholine, which is required for the synthesis of new phosphatidylcholine (PC). Choline has an affinity for fat. On the one hand, it synthesizes lecithin and transports fat from the liver in the form of lipoproteins, thereby reducing the deposition of fat in the liver. On the other hand, choline can increase the oxidative metabolism of fatty acids in the liver, preventing the abnormal accumulation of fat in the liver and demonstrating an anti-fatty liver effect. As a non-neural cell intercellular signaling molecule, non-neurogenic Ach participates in the regulation of the signal transmission and communication of endocrine hormones and growth factors in these cells and the surrounding microenvironment. It is significantly different from neuronal Ach. For example, non-neurogenic Ach has the effect of promoting the proliferation of pre-adipocytes and the release of pro-inflammatory factors. Studies have found that both white preadipocytes and mature adipocytes express Ach esterase AChE, Ach transferase ChAT, and Ach receptor AchR. The choline level found in this study was significantly reduced in the obese and HLO groups, which may be due to increased metabolism or decreased synthesis of Ach in the body when the intake of exogenous choline remains unchanged.

Glutamine (Gln), a non-essential amino acid in human plasma, mainly used for energy production, is synthesized in the skeletal muscles and hepatocytes and is released into the blood circulation [40]. Lower glutamine in circulation shows lower energy expenditure and is lower in lean mass construction, which subsequently lowers the metabolic rate at rest and thus excess energy tends to be stored as ectopic fat deposits, especially in the intraabdominal part. The existing mass spectroscopic (MS)-based metabolomics study also showed that serum glutamine concentration is lower in obese children than their normal-weight counterparts [41]. We also found a lower glutamine level in both OW/OB and HLO compared with NW and NHLO groups, indicating that the lower glutamine levels are associated with larger adipocyte sizes and higher body fat percentages, not only BMI. In addition, we confirmed the presence of significantly lower concentrations of glutamine and glucose in the serum of HLO subjects. Even though the metabolic mechanisms involved in glutamine levels that result in a reduction in HLO have not yet been clarified, lower glutamine levels could point to the fact that obesity can be associated with hyperlipidemic situations.

Certainly, glutamine is the main metabolite mediator of the hexosamine pathway, a minor branch of glycolysis. Fructose-6-phosphate in glycolysis is converted to glucosamine-6-phosphate and is catalyzed by glutamine: fructose-6-phosphate aminotransferase (GFAT). It is rate-limiting enzyme and is the first enzyme that is released from utilization of glutamine that enters into the cell and degrades to glutamate [42]. This is why the greater activation of the hexosamine pathway in the obese demonstrates a higher anabolism of glutamate metabolites. Glutamate, a by-product of BCAA catabolism, had proportional increases in visceral fat content in this study. Yakamado et al. assessed VAT by computed tomography (CT) in 1449 Japanese subjects, and found that glutamate was the single metabolite most strongly correlated with VAT area (r = 0.49, *p* < 0.001) [43]. Our results strongly supported this fact in both OW/OB and HLO groups with the visceral fat % correlation coefficient (r = 0.562, *p* < 0.001). Moreover, we found a lower level of glucose (increased glucose consumption) and higher N-acetyl due to a subsequent result of hyperactivation of the hexosamine pathway especially in hyperlipidemic conditions. In glycoproteins or N-acetyl glycoprotein, this signal is produced by the COCH_3_ acetyl groups of N-acetylglucosamine, N-acetylgalactosamine and N-acetylneuraminic acid [44,45]. This factor is entirely in agreement with an NMR-based study done on Ningxiang growing pigs that also showed lower glucose levels combined with higher N-acetyl glycoproteins in the plasma of obese pigs compared with lean pigs [46,47]. Altogether, these findings suggest that N-acetyl glycoproteins might be an early marker in obesity-related hypercholesterolemia caused by hexosamine pathway activation. In other words, N-acetyl glycoproteins can be a cardiovascular predictor in linking hyperlipidemia obesity to metabolic deterioration.

Acetoacetate, the principal ketone body, is synthesized mainly in the liver when acetyl CoA overspills into the body, whenever glucose does not have enough energy, or when tricarboxylic acid (TCA) cycle processes are failing. The liver converts about 60% of those acetyl CoAs derived from fatty acid oxidation and catabolism of some amino acids into ketone bodies during ketogenesis. The TCA cycle is the central hub designed to produce body energy from carbohydrates, fats, and protein metabolism. The significant decrease that occurred in TCA cycle intermediates in the urine and feces of HFD induced obese rats demonstrated that the TCA cycle is suppressed in an obese state, and this NMR report is consistent with previous literature [31,48]. Furthermore, Daxesh P. Patel et al. proved that the downregulation of TCA cycle activity in HFD-fed mice was due to anaplerosis impairment by omics single-ion monitoring (SIM) in a GCMS study on both serum and liver extract samples [49]. The suppression of the TCA cycle indicates a decrease in the energy consumption rate in an obese state. Thus, the surplus energy is stored as ketone bodies or as ectopic fat accumulation in different locations such as intrahepatic, intraabdominal, and upper-end thigh regions. In this study, the serum acetoacetate level was significant and the highest change percentage of 45.45% occurred between NHLO and HLO but was not obviously between NW and OW/OB groups. However, the biochemically stable and reduced form of acetoacetate, 3 hydroxybutyrate, is significantly higher in OW/OB than NW. Therefore, the serum ketone level elevation signifies that a tissue uses fat for energy instead of glucose under conditions of obesity. (Therefore, the increase of serum ketone level may be due to the fact that the energy consumed by peripheral tissues of obese individuals is more from fat than glucose). This result shows that the primary ketone substance, acetoacetate, may be the strongest factor for HLO and the acetoacetate derivative 3 hydroxybutyrate can be used to distinguish OW/OB from NW.

Leucine, isoleucine, and valine are collectively referred to as BCAAs due to their shared structural features in the side-chain and a common catabolic pathway. Previous studies have reported strong associations between increased BCAA levels, obesity, and metabolic diseases such as type 2 diabetes [50]. A study done on both rodents and human trials showed that BCAA serum concentrations were significantly higher among obese subjects than among lean ones [51,52]. Mass spectroscopy studies on plasma BCAA levels in humans, led to a significant increase that was observed among OW/OB compared to NW individuals. Furthermore, when MetS status is considered, OW/OB individuals with MetS have a higher amount of each BCAA compared to those obese participants without MetS [36]. Our results agreed with those findings, and in addition, isoleucine had a more significant *p*-value, greater incremental percentages, and increases of VIP scores in OW/OB compared to NW and HLO relative to NHLO groups. Moreover, isoleucine was positively and moderately associated with metabolically active VAT content. This association was only observed whenever the significant correlations between WC and isoleucine were seen among OW/OB subjects with and without MetS.

The results will help to develop personalized prevention and treatment methods for obesity. We propose prevention and treatment strategies based on the different mechanisms and phenotypic characteristics of NHLO and HLO. For example, this could be for obese patients whose blood lipid metabolism disorders have not yet appeared (NHLO), so it is mainly aimed at treating adipocyte differentiation abnormalities and over accumulation of lipids in the white adipocytes and the hepatocytes. Obese people with serum lipid metabolism disorders (HLO) need to be treated using the countermeasures listed above for further treatment, such as those based on finding the causes of abnormal blood lipid metabolism and adipocyte differentiation so that dual or multiple prevention and treatment strategies can be applied.

## 5. Conclusions

This presented ^1^H NMR study reveals that the OW/OB serum metabolome is clearly different from the normal-weight serum metabolome. Obese subjects show higher lipid concentrations, indicating that the hepatic production and secretion of triglycerides are increased. A higher blood lactate level in obesity shows the association of low mitochondria functioning adipocytes with activation of the anaerobic glycolysis pathway for ATP synthesis. Lower levels of glutamine and glucose, and higher levels of N-acetyl glycoprotein and glutamate, could point to hyper-activation of the hexosamine pathway of the obese under hyperlipidemia conditions. Increased acetoacetate and 3-hydroxybutyrate in the obese serum can be linked to an impaired tricarboxylic acid cycle, reflecting a disturbed energy metabolism. As expected, a lower level of phosphatidylcholine, representative of choline, which was observed in obese adolescents, was suggested to be associated with the development of obesity and metabolic syndrome. BCAAs (valine, isoleucine, and leucine) were found to be higher in the serum of obese as compared with normal-weight adolescents, which might point to the likelihood that BCAA levels could be used as a predictor for future metabolic diseases among OW/OB people.

This study reveals valuable findings regarding metabolomics and young adult obesity and associated hyperlipidemia. We propose several serum biomarkers that distinguish between normal-weight and obese adults. Glutamine was higher in normal-weight, and lactate, BCAAs, acetoacetate and 3-hydroxybutyrate were higher in the obese. In addition, visceral fat and serum TG, glutamate, acetoacetate, N-acetyl glycoprotein, unsaturated lipid, isoleucine and VLDL/LDL were significantly higher in the obese with hyperlipidemia. Therefore, these compounds could be used as biomarkers to identify these two types of obesity.

Moreover, metabolites indicate the characteristics and differences between hyperlipidemia and non-hyperlipidemia in obese subjects. These results will help to develop personalized prevention and treatment methods for obesity.

## Figures and Tables

**Table 1:** Descriptive characteristics of biophysical and biochemical profiles between the NW group and OW/OB group.

Parameters	NW	OW/OB	*p*-Value
Age	21.50 ± 0.63	21.50 ± 0.95	0.981
Weight (kg)	53.80 ± 4.68	83.29 ± 12.17	<0.001
Height (cm)	161.61 ± 1.93	168.64 ± 8.65	<0.001
BMI (kg/m^2^)	20.60 ± 1.64	30.48 ± 4.37	<0.001
WC (cm)	69.57 ± 4.24	96.70 ± 11.76	<0.001
HC (cm)	91.03 ± 2.67	110.67 ± 9.02	<0.001
TC (mg/dL)	193.47 ± 18.03	210.47 ± 36.67	<0.05
TG (mg/dL)	72.50 ± 13.25	115.23 ± 43.89	<0.001
HDL-C (mg/dL)	56.60 ± 7.95	50.30 ± 9.47	<0.05
LDL-C (mg/dL)	119.45 ± 14.60	133.79 ± 27.33	<0.05
SGPT(ALT) U/L	10.40 ± 2.40	24.26 ± 12.89	<0.001
FBS (mg/dL)	82.97 ± 2.71	89.45 ± 5.21	<0.001
HbA1c %	5.01 ± 0.195	5.19 ± 0.17	<0.001
Visceral fat %	3.28 ± 1.01	11.30 ± 4.31	<0.001

All data are presented as mean and standard deviation values. NW, normal-weight group. OW/OB, overweight/obese group.

**Table 2:** Serum variables resolved by ^1^H NMR of NW group and OW/OB group.

No.	Assigned Metabolites	ppm, δ	NW	OW/OB	*p*-Value	Trend	Change %	VIP
			Mean ± SD	Mean ± SD				
1	Unsaturated lipid (CH=CH)	5.3	0.029 ± 0.003	0.033 ± 0.006	<0.05	↑	13.11	1.01
2	Alpha glucose	5.23	0.016 ± 0.001	0.017 ± 0.002	0.07	↑	5.42	0.878
3	Beta glucose	4.63	0.016 ± 0.002	0.017 ± 0.002	<0.05	↑	8.62	0.985
4	Lactate	4.1	0.006 ± 0.001	0.008 ± 0.002	<0.001	↑	39.93	0.765
5	Total glucose	3.35 −3.92	0.328 ± 0.027	0.348 ± 0.037	<0.05	↑	5.97	0.722
6	TMAO	3.25	0.013 ± 0.001	0.013 ± 0.002	0.854	↑	0.545	0.743
7	Carnitine	3.23	0.019 ± 0.002	0.019 ± 0.002	0.434	↑	2.68	0.832
8	Choline	3.21	0.068 ± 0.009	0.049 ± 0.011	<0.001	↓	−28.54	1.58
9	Creatine	3.04	0.004 ± 0.000	0.004 ± 0.001	0.812	↑	1.39	0.570
10	Glutamine	2.45	0.010 ± 0.001	0.009 ± 0.002	0.052	↓	−8.98	0.997
11	Glutamate	2.34	0.004 ± 0.001	0.006 ± 0.002	<0.001	↑	47.52	1.35
12	Acetoacetate	2.22	0.003 ± 0.001	0.003 ± 0.001	0.228	↑	10.24	0.789
13	N-acetyl glycoprotein	2.14	0.001 ± 0.00	0.002 ± 0.000	<0.05	↑	17.69	0.678
14	Unsaturated lipid (=CH_2_)	2.02	0.054 ± 0.004	0.067 ± 0.009	<0.001	↑	24.41	1.86
15	Lysine	1.91	0.001 ± 0.000	0.001 ± 0.000	0.70	↑	2.34	0.433
16	Alanine	1.48	0.012 ± 0.001	0.013 ± 0.002	<0.001	↑	15.94	0.582
17	Lactate	1.3	0.049 ± 0.007	0.061 ± 0.017	<0.001	↑	26.41	1.14
18	(-CH_2_)_n_ VLDL/LDL	1.27	0.119 ± 0.017	0.157 ± 0.038	<0.001	↑	31.97	1.52
19	3 hydroxybutyrate	1.17	0.008 ± 0.001	0.009 ± 0.003	0.05	↑	19.69	0.455
20	Valine	1.047	0.007 ± 0.001	0.008 ± 0.001	<0.05	↑	13.35	0.886
21	Isoleucine	1.02	0.002 ± 0.000	0.003 ± 0.001	<0.001	↑	23.77	1.13
22	Valine	0.996	0.010 ± 0.002	0.012 ± 0.002	<0.05	↑	14.57	0.993
23	Leucine	0.96	0.012 ± 0.002	0.015 ± 0.004	<0.05	↑	19.95	0.587
24	(-CH_3_) VLDL/LDL	0.87	0.127 ± 0.009	0.132 ± 0.013	0.095	↑	3.876	0.977
25	Total lipid		0.330 ± 0.033	0.390 ± 0.058	<0.001	↑	18.20	

Calculated using independent two samples t-test and presented as mean and SD (standard deviation). Significant values are shown as *p*-value < 0.05 and <0.001. % change is the increase (+) or decrease (−) of the mean in the OW/OB group with respect to the NW group. NW, normal-weight group; OW/OB, overweight/obese group, and VIP, variable important on projection. VLDL, very low-density lipoprotein; LDL, low-density lipoprotein; TMAO, trimethylamine N-oxide.

**Table 3:** Pearson’s correlation of ^1^H NMR lipid metabolites with biochemical lipid profiles.

No.	Metabolites	Correlation with -CH=CH Lipid	Correlation with =CH_2_ Lipid	Correlation with (-CH_2_)_n_ VLDL/LDL	Correlation with CH_3_ VLDL/LDL	Correlation with Lactate (1.3 ppm)
		r	r	r	r	r
1	TC (mg/dL)	0.422 *	0.423 *	0.453 **	0.490 **	0.360 *
2	TG (mg/dL)	0.462 **	0.451 **	0.485 **	0.570 **	0.408 *
3	HDL-C (mg/dL)	−0.206	−0.245	−0.248	−0.177	−0.305 *
4	LDL-C (mg/dL)	0.424 *	0.400 *	0.433 **	0.486 **	0.354 *
5	ALT (U/L)	0.313 *	0.324 *	0.339 *	0.343 *	0.231

All data are presented as ‘r’ values of Pearson linear correlation. Significant values are shown as * *p* < 0.05, ** *p* < 0.001. TC, total cholesterol; TG, triglyceride; HDL-C, high-density lipoprotein; LDL-C, low-density lipoprotein and ALT, alanine transaminase.

**Table 4:** Pearson’s correlation of ^1^H NMR glucose metabolites with biochemical glucose profiles.

No.	Metabolites	Correlation with Alpha Glucose	Correlation with Beta-Glucose	Correlation with Total Glucose	Correlation with Lactate (1.3 ppm)
		r	r	r	r
1	HbA1c %	0.243	0.631 **	0.310 *	0.525 **
2	FBS (mg/dL)	0.050	0.487 **	0.134	0.529 **

All data are presented as ‘r’ values of Pearson linear correlation. Significant values are shown as * *p* < 0.05, ** *p* < 0.001. HbA1c, glycated blood glucose, and FBS, fasting blood sugar.

**Table 5:** Pearson’s correlation of ^1^H NMR metabolites with visceral fat content percentage.

No.	Metabolites	Correlation with Visceral Fat %	
		r	*p*-Value
1	Unsaturated lipid (CH=CH)	0.433	<0.001
2	Choline	−0.626	<0.001
3	Glutamate	0.562	<0.001
4	Unsaturated lipid (=CH_2_)	0.656	<0.001
5	Lactate (1.3 ppm)	0.372	<0.05
6	(-CH2)_n_ VLDL/LDL	0.561	<0.001
7	Isoleucine	0.419	<0.05

All data are presented as ‘r’ values of Pearson linear correlation. Significant values are shown as *p* value < 0.05 and <0.001.

**Table 6:** Descriptive characteristics of biophysical and biochemical profiles between non-hyperlipidemia obesity (NHLO) and hyperlipidemia obesity (HLO) groups.

Parameters	NHLO	HLO	*p*-Value
Age (years)	21.6 ± 1.1	21.1 ± 1.5	0.311
Weight (kg)	81.6 ± 14.9	84.8 ± 9.8	0.541
Height (cm)	167.12 ± 8.11	168.85 ± 8.91	0.609
BMI (kg/m^2^)	30.27 ± 4.65	31.18 ± 4.22	0.607
WC (cm)	96.69 ± 11.56	97.08 ± 12.43	0.936
HC (cm)	112.23 ± 9.06	111.38 ± 8.53	0.808
TC (mg/dL)	183.92 ± 16.89	238.77 ± 33.67	<0.001
TG (mg/dL)	92.46 ± 38.97	138.69 ± 42.17	<0.05
HDL-C (mg/dL)	48.23 ± 10.97	50.85 ± 8.45	0.503
LDL-C (mg/dL)	117.2 ± 16.58	160.18 ± 3 2.12	<0.001
SGPT(ALT) U/L	23 (14.5–45)	22 (14–39.5)	0.797
FBS (mg/dL)	90 (81.5–93.5)	88 (86–91.5)	0.719
HbA1c %	5.21 ± 0.256	5.25 ± 0.194	0.67
Visceral fat %	8.77± 3.29	13.83± 3.75	<0.05

**Table 7:** Serum variables resolved by ^1^H NMR of non-hyperlipidemia obesity (NHLO) and hyperlipidemia obesity (HLO) groups.

No.	Assigned Metabolite	ppm, δ	NHLO	HLO	*p*-Value	Trend	Change %	VIP
1	Unsaturated lipid (CH=CH)	5.3	0.030 ± 0.005	0.037 ± 0.005	<0.05	↑	25.49	0.831
2	Alpha glucose	5.23	0.017 ± 0.001	0.016 ± 0.002	0.050	↓	−8.09	0.535
3	Beta glucose	4.63	0.018 ± 0.002	0.017 ± 0.002	0.154	↓	−6.27	0.902
4	Lactate	4.1	0.007 ± 0.001	0.008 ± 0.002	0.481	↑	7.17	0.712
5	Total glucose	3.35–3.92	0.356 ± 0.028	0.329 ± 0.036	0.059	↓	−7.77	1.071
6	TMAO	3.25	0.013 ± 0.001	0.013 ± 0.002	0.936	↓	−0.47	0.826
7	Carnitine	3.23	0.019 ± 0.002	0.019 ± 0.003	0.824	↑	1.18	1.161
8	Choline	3.21	0.054 ± 0.012	0.043 ± 0.005	<0.05	↓	−20.65	0.878
9	Creatine	3.04	0.004 ± 0.001	0.005 ± 0.001	0.324	↑	12.04	1.142
10	Glutamine	2.45	0.010 ± 0.002	0.008 ± 0.001	<0.05	↓	−21.02	0.944
11	Glutamate	2.34	0.006 ± 0.001	0.007 ± 0.002	<0.05	↑	23.75	1.332
12	Acetoacetate	2.22	0.003 ± 0.001	0.004 ± 0.001	<0.05	↑	45.45	1.362
13	N-acetyl glycoprotein	2.14	0.001 ± 0.000	0.002 ± 0.000	<0.05	↑	32.31	1.185
14	Unsaturated lipid (=CH_2_)	2.02	0.063 ± 0.008	0.072 ± 0.006	<0.05	↑	15.76	1.406
15	Lysine	1.91	0.001 ± 0.000	0.001 ± 0.000	0.985	↑	0	0.643
16	Alanine	1.48	0.013 ± 0.002	0.014 ± 0.002	0.322	↑	7.16	1.021
17	Lactate	1.3	0.054 ± 0.013	0.068 ± 0.015	<0.05	↑	25.78	0.650
18	(-CH_2_)_n_ VLDL/LDL	1.27	0.132 ± 0.024	0.185 ± 0.030	<0.001	↑	40.02	0.667
19	3 hydroxybutyrate	1.17	0.008 ± 0.001	0.010 ± 0.004	0.205	↑	18.80	0.588
20	Valine	1.047	0.008 ± 0.001	0.008 ± 0.001	0.781	↑	1.67	1.066
21	Isoleucine	1.02	0.003 ± 0.001	0.003 ± 0.000	<0.05	↑	18.75	1.287
22	Valine	0.996	0.011 ± 0.002	0.012 ± 0.001	0.467	↑	4.46	0.983
23	Leucine	0.96	0.014 ± 0.003	0.016 ± 0.004	0.206	↑	15.35	0.914
24	(-CH_3_) VLDL/LDL	0.87	0.125 ± 0.008	0.141 ± 0.012	<0.05	↑	12.11	1.136
25	Total lipid		0.363 ± 0.046	0.417 ± 0.058	<0.05	↑	14.91	

Calculated using independent two samples t-test and presented as mean and SD (standard deviation). Significant values are shown as *p*-value, % change is the increase (+) or decrease (−) of the mean in the HLO group with respect to the NHLO group. NHLO, non-hyperlipidemia obese group; HLO, hyperlipidemia obese group; VIP, variable important on projection; VLDL, very-low-density lipoprotein; LDL, low-density lipoprotein; TMAO, trimethylamine N- oxide.
